# The biophysical bases of will-less behaviors

**DOI:** 10.3389/fnint.2012.00098

**Published:** 2012-10-23

**Authors:** José L. Perez Velazquez

**Affiliations:** ^1^Neuroscience and Mental Health Programme, Division of Neurology, Brain and Behaviour Centre, Hospital for Sick Children, University of TorontoToronto, ON, Canada; ^2^Department of Paediatrics and Institute of Medical Science, University of TorontoToronto, ON, Canada

**Keywords:** brain synchrony, coherence, neurophysics, free will, volition, excitability, psychiatry, brain coordination dynamics

## Abstract

Are there distinctions at the neurophysiological level that correlate with voluntary and involuntary actions? Whereas the wide variety of involuntary behaviors (and here mostly the deviant or pathological ones will be considered) will necessarily be represented at some biophysical level in nervous system activity–for after all those cellular activity patterns manifest themselves as behaviors and thus there will be a multiplicity of them–there could be some general tendencies to be discerned amongst that assortment. Collecting observations derived from neurophysiological activity associated with several pathological conditions characterized by presenting will-less actions such as Parkinson's disease, seizures, alien hand syndrome and tics, it is proposed that a general neurophysiologic tendency of brain activity that correlates with involuntary actions is higher than normal synchrony in specific brain cell networks, depending upon the behavior in question. Wilful, considered normal behavior, depends on precise coordination of the collective activity in cell ensembles that may be lost, or diminished, when there are tendencies toward more than normal or aberrant synchronization of cellular activity. Hence, rapid fluctuations in synchrony is associated with normal actions and cognition while less variability in brain recordings particularly with regards to synchronization could be a signature of unconscious and deviant behaviors in general.

## Introduction

The main query to be addressed here is whether there are special patterns of activity in the nervous system, particularly in the brain, that are associated with wilful and will-less behaviors. Based on what is known regarding the correlation of certain neurophysiological activity patterns with pathological or deviant behaviors, it is tempting to conclude that there seems to be a clear tendency in brain collective dynamics that result, or manifest themselves, as will-less actions. This article explores evidence that long-lasting widespread synchronization in specific brain networks results in behavioral deviations including will-less actions.

The underlying general principles could be relatively simple in spite of mechanistically complicated processes. However, as is common in scientific endeavors, a dichotomy is lurking here, the dualistic vision of intention and behavior, one of the oldest and most debated dualisms in the history of science: mind versus brain. Nevertheless, it is feasible to transcend that duality, to set aside the dichotomy (which in the end could be beneficial to neuroscience, as centuries of discussion on the mind-body “problem” has not resulted in any apparent satisfactory solution). Thus, questions about the causal role of intentions on behavior acquire a different perspective from which improved unbiased answers can be obtained.

Take a look for instance at the following enquiry into a well-known relation between a brain specific activity and behaviors: as found out many decades ago, the μ rhythm, an oscillation of 8–12 Hz recorded mostly from central sensory-motor cortical areas, tends to diminish or disappear when the subject executes, visualizes, or thinks about executing movements (motor imagery). This rhythm was thus associated with intentional actions and it has been recently shown to correlate with intentional social coordination as well (Naeem et al., 2012). One can then ask, in a dichotomy-minded setting, whether the change in this specific activity pattern, the μ oscillation representing a collective activity in millions of brain cells, is causing the behavior, or whether it is the behavior that brings about the temporary demise of this brain rhythm. However, this question loses, or perhaps acquires a different meaning, if both the neurophysiological activity (the μ rhythm) and the behavior are considered aspects of the same phenomenon: the collective activity patterns manifest themselves as actions, and actions in turn are those coordinated patterns; the only difference consists of the tissues involved. In overt actions muscles and bones take part, indeed extending the collective coordinated activity to a multi-system level including the nervous and the skeletal systems. This style of thought has the flavor of a loop that may not be welcome by some, but loops are omnipresent in biology, and cognition in particular (Hofstadter, 1979). This non-dualistic frame of mind is beneficial for a better comprehension of the topic addressed here and the remarks that will follow in subsequent sections.

The central argument is that widespread long-lasting synchronous activity amongst brain regions disrupts the transient coordination for optimal, adaptive behavior. That a certain degree of coordination amongst the activity of cell ensembles in the nervous system is needed for proper behaviors (and information processing in general) is well known from the invertebrates (think of the cellular activity in a central pattern generator that produces different oscillatory patterns that manifest as varied movements of the animal depending on the relation of those cellular firing patterns) to vertebrates; and that cell assemblies organize their activity through transient synchronization has been proposed by many investigators (von der Malsburg, 1981; Varela et al., 2001; Singer, 2006; Kelso, 2008). The consequences of altered interactions amongst brain oscillations are multiple but in the final analysis it can be described using synchrony measures (Schnitzler and Gross, 2005). The coordination dynamics of nervous systems is reflected in synchrony patterns but in the case of higher brains (as opposed to older structures such as central pattern generators) these patterns are very transient and a certain degree of variability in synchrony is expected for proper and adaptive cognition to develop. More widespread and long-lasting synchrony may disrupt the “healthy” coordination dynamics with the consequent appearance of abnormal behaviors (Perez Velazquez and Frantseva, 2011). Thus, cellular hyperexcitability increases the tendency toward long-lasting widespread synchronization in the brain resulting in behavioral deviations including will-less actions, a notion that has received experimental support at the molecular/cellular level in a recent study. Using optogenetic tools, Yizhar et al. (2011) altered the balance between excitation and inhibition in mouse brains (specifically prefrontal areas) and found that more excitation resulted in impairment of information processing in general and in abnormal social behaviors (these being social exploratory actions that mice perform), and these behavioral alterations were concomitant with enhanced rhythmic activity (a sign of increased synchrony). Restoring excitation-inhibition by enhancing in these mice, restored the normal behaviors.

Along the same lines, Yang et al. (2012), using *in vitro* organotypic rat cortical slices, reported that an optimal level of synchrony (here optimality defined as that which does not create paroxysms, or neuronal avalanches as these are termed in the paper) is associated with a narrow range of excitation-inhibition ratio and furthermore it coincides with maximal variability in synchrony, that the authors interpret as states being near criticality, where the system can benefit from moderate (“healthy”) levels and maximal variability of synchrony.

Thus, these two recent studies provide more detailed mechanistic support for the notion that hyperexcitability enhances the probability to widespread, less variable and long-lasting synchrony that results in behavioral deviations and neurological pathologies. If this were to be the case in general, the job for clinicians in different specialties is to discover the areas in the nervous system (does not have to be restricted to the central) of more-than-normal activity and synchrony in each patient and target those areas with specific interventions to reduce the pronounced tendency of all nervous cells to synchronize activity. This is obviously easier said than done, as a localized damage may produce alterations of function in remote connected areas (think of diaschisis for instance), but this could be a common framework to understand most neural pathologies.

## A cautionary note on “connectivity”

Before proceeding, some preliminary, technical comments on the notions of synchronization and connectivity are needed. Those who follow the literature will have noticed the variety of distinct results in “brain connectivity” analysis: task-related connectivity studies yield different results from resting (no task), and sometimes even identical tasks or apparently same resting conditions have resulted in an assortment of connectivity patterns. Why this variability? In what follows the word “connectivity” or its close relatives like “synchrony” shall appear several times, and while most of neuroscientists know what these terms denote, best to clarify a few points on these matters now at the start of this narrative.

Firstly, some disorders such as schizophrenia or autism have been classically labeled as syndromes of “dysconnectivity” at the brain physiological level, categorization that originally stems from anatomical and psychological findings mostly. However, it is a different matter to more directly assess or compute dynamic “connectivity” amongst brain areas using synchrony or graph theory analysis, to cite two common analytical methods widely used these days. Thus, the apparent disconnection in schizophrenia becomes a *different* type of connectivity when methods such as graph theory are used, in that there is a shift in the schizophrenic brain from local to more global connectivity (Rubinov et al., 2009; van den Berg et al., 2012). Similar comments can be said about autism studies, where rather than disconnection what emerges is a different pattern of synchronization (Perez Velazquez et al., 2009; Teitelbaum et al., 2012).

The second consideration is the fact that enhanced connectivity between two brain areas is observed from physiological signals such as the electroencephalogram or functional neuroimaging data, does not necessarily mean that those areas are more synchronized in their activities. In fact, it does not even imply a stronger real connectivity, because most of the connectivity analyses performed these days evaluate *correlations* in activity, and if two areas display correlated activity then the assumption is made they are “strongly connected,” but this may or may not be the case. Correlations in activity between two systems (brain regions) can be due to several phenomena and not necessarily via direct mutual interactions (they could be driven by a third source for instance). These topics have been discussed previously (Perez Velazquez and Frantseva, 2011) and this is not the time to delve on these matters, suffice to mention that changes in connectivity will be taken, in this work, just as indicators of differences in coordinated/correlated activity, and especially in synchronization.

Let us now proceed to consider some involuntary actions and their associated neurophysiological activity patterns.

## Automatisms during seizures

Perhaps the most prominent characteristic examples of what apparently are involuntary actions occur during seizures in epileptic patients, automatisms that many times are well-coordinated behaviors such as picking up objects. Notwithstanding the controversy about the extent to which subjects lose consciousness during seizures (Gloor, 1986; Blumenfeld and Taylor, 2003), at least during complex partial seizures, it is very probable that the actions remain mostly involuntary during ictal events. The main reason for this controversy is that the level of awareness during seizures cannot be known because patients are not responsive hence communication at the moment of the brain paroxysmal event is not feasible. The answers obtained after the ictal event indicate that patients are normally unaware of the actions during seizures but this could be due to improper formation of the memory trace (hence from the patient's viewpoint the event never existed) due to the altered brain activity characteristic of the ictus. Nevertheless, if we assume that the actions are involuntary, then the underlying neurophysics consists in the paroxysmal activity in extensive cell populations: prominent increase in cellular excitability and synchronization.

The nature of these paroxysmal discharges, (the electrographic seizures), can be encapsulated in two main aspects: higher than normal excitability in nervous tissue leading to extended periods of synchronization. Details about specific cellular and molecular aspects of epileptiform activity can be found in many texts (Jefferys, 1990; McCormick and Contreras, 2001; Perez Velazquez and Wennberg, 2004). For the present purposes, the important matter is that the abnormal synchrony patterns during seizures result in profound alterations of the coordinated activity of cerebral networks with the consequent impairment of information processing, and thus, unawareness of the ictal automatisms could possibly result.

At the root of the phenomenon then two tendencies are found common to many pathologies: the tendency to increase excitability as well as synchrony in specific brain regions. There is wide empirical evidence for both of these phenomena, (hyperexcitability and higher than normal synchronization), during seizure activity (reviewed in chapter three of Perez Velazquez and Frantseva, 2011). It should perhaps be noted here, for fairness' sake, that there are a few studies reporting lower than normal synchrony during seizures. Nevertheless, different results on synchrony can be obtained if synchronization is evaluated between collective activities of cellular ensembles (like local field potentials representing mostly synaptic potentials), or between individual spike firing in neurons so that desynchronization in the spike firing of individual neurons (this would be at short time scales, or high frequencies) and synchronization at the slower time scale (low frequencies) of bursting activity and synaptic inputs can occur simultaneously. It is conceivable that there could be differences in coordinated activity at several levels so there is no reason to despair at the apparently different and opposite reports that appear in the literature, it all depends on the level of description and methods used. What is apparent is that there are abnormal patterns of synchronized activity associated with epileptiform activity.

Hyperexcitability before and during the seizure is not as controversial as the aforementioned studies on the degree of synchrony. In fact, already three decades ago, Babb et al., using chronically implanted microelectrodes into areas of the limbic system in patients, determined individual spike firing (unit activity) in neurons, and their results showed that there is a population of neurons that depolarize simultaneously and are more prone to discharge synchronously, and that the number of these neurons that increase the firing rates determine the severity and extent of the seizure propagation. For example, they estimated that about 7% of the recorded neurons increased the firing rates during sub-clinical seizures (these are seizures that do not alter consciousness and have no behavioral manifestations, thus the term “sub-clinical”), whereas about 14% of neurons augmented the firing rates during auras (specific sensations patients develop just before the ictal event, even though there are those who consider auras as part of the seizure), and 36% of neurons during clinical seizures (Babb et al., 1987). Thus, this early study demonstrates that seizure severity is accompanied by enhanced cell firing. The observation of an increase in BOLD signal ~5 s prior to seizure onset (Bai et al., 2010) is a further indication of greater excitability occurring before the synchronous activity becomes apparent in the recordings, and along the same line it has been known for decades that high frequency activity is detected at the onset of ictal events (Fisher et al., 1992). Nowadays, there seems to be a re-surge of this long-known notion denoted by new expressions such as high frequency oscillations (HFOs), upon which there is an abundant, ever-increasing literature.

In summary, the most probable scenario, considering the many studies on these topics, indicate an increased synchronized activity during seizures that will involve distinct brain structures and, depending on where this abnormal coordinated activity occurs, different behaviors (automatisms) can be manifested determined by the chains of brain cells involved. As well, conditional on the extent and magnitude of this synchronization, different degrees of paroxysms can be expected. For instance, one unusual automatism in epileptic patients is to perform the sign of the cross. Wennberg et al. (2009) reported the case story of a patient who apparently was unconscious during seizures and involuntarily made the signum crucis, an action that, interestingly, the patient had performed voluntarily after the ictal events, following her parents' advice, early in her disease (seizures were seen in the past as diabolical interventions). The fact that now the action is involuntary suggests that the neural activity of her seizures has integrated the neural networks (motor areas and others) that were responsible for that specific and at early time voluntary act, so now the action occurs during the ictus and it remains out of consciousness. This intriguing case provides food for thought as it reflects the integration of neurophysical and psychological levels and illustrates how brain ensembles of cells “storing” certain actions can be recruited by paroxysms or aberrant synchronous activity and yet may remain out of mind because the abnormal coordination dynamics during these events perturb the integration and segregation of information characteristics of normal brain function.

## Passivity syndromes

Perhaps related to the phenomenon of higher synchronous activity associated with involuntary actions, it has been known for some time that dissociative states (multiple personality disorders as they were called in past times) occur in patients with “abnormal” temporal lobe EEG that is similar to temporal lobe seizures. Mesulam (1981) describes seven of these patients with dissociative states and five others that experienced illusion of external possession, and the EEG recordings of these individuals presented paroxysmal events even though these were not always correlated in time with the psychiatric episodes. Nevertheless, these types of will-less behaviors were associated with abnormal (higher than normal in those cases) brain synchronization.

Further evidence for more synchrony in brain activity during involuntary actions comes from a study of schizophrenic patients with passivity phenomena. Passivity delusions involve the belief that the actions are influenced by external agents and are perceived as involuntary to some extent. These patients displayed hyperactivation of parietal and cingulate cortices (areas fundamental for attention to and programming of motor actions in space) that decreased over time in those patients that showed improved (that is, diminished) passivity phenomena. Hyperactivity in neurons greatly increases the probability of “hypersynchrony,” as expounded in Perez Velazquez and Frantseva (2011). Schizophrenics without passivity symptoms did not display the hyperactivation seen in the other patients, nor control subjects (Spence et al., 1997). In general, it could be concluded from the current evidence that there could be a tendency of the schizophrenic brain to display more synchronized activity with less variability in the coordinated activity patterns. What Lee et al. (2003) termed overbinding: abundance of random connections such that distinguishing internal from external inputs becomes problematic and thus hallucinatory phenomena may appear.

## Disinhibition syndromes

Related to automatisms during periods of high synchronization, the following examples of involuntary actions occurring not during clinical seizures but nonetheless, are associated with paroxysmal activity and are reflections of the dis-coordination of brain cell ensembles due to the hyperexcitability and hypersynchrony commented above. Thus, another illustration of involuntary acts is the rare neurological disorder known as alien hand syndrome. Individuals suffering from this symptom claim that the actions of, normally, one of their hands are controlled by other forces that do not depend on them, thus the actions of those limbs can be regarded as will-less. It could be argued that patients still perceive/sense/feel the alien actions as being intentional and this is why they think their limbs are controlled by other entities, for in the final analysis will is, like everything else, a perception, as Hume noted in his “Treatise of Human Nature” of 1739: “ … that by the will I mean nothing but the internal impression we feel and are conscious of, when we knowingly give rise to any new motion of our body, or new perception of our mind.” Still, there is a grade of involuntariness in these patients' actions and this is not an all-or-none phenomenon.

While most studies about this syndrome have been neuropsychological or clinical in terms of finding brain lesion sites, almost nothing is known at the neurophysiological level. However, there is a case report that found paroxysmal activity in discrete cortical areas recorded with scalp EEG that correlated with alien hand episodes (Brázdil et al., 2006). Another case report, using functional MRI, found brain widespread activity when the movements were voluntary but only localized, restricted activity in the contralateral primary motor area (M1) during alien hand episodes (Assal et al., 2007), therefore indicating that intentionality requires the coordinated activity of widespread brain networks. This notion finds support in the global workspace theory, in that extensive broadcast of information throughout many brain areas is required for attention and awareness in general (Baars, 1988), and emphasizes the importance of the proper coordinated activity amongst varied and extended brain cell ensembles for purposeful behavior to occur. Distortions of that coordination either by more (or less) than the necessary synchronization will favor non-awareness and the emergence of deviant behaviors.

Another example where actions are driven by external stimuli and inhibition seems lost is that of utilization behavior. Individuals suffering from utilization behavior (and the related imitation behavior originally described by Lhermitte in 1983 as having environmental dependency syndrome), have difficulty resisting the impulse to operate or manipulate objects which are in their visual field and within reach. Characteristics of this syndrome include unintentional actions triggered by the immediate environment, and therefore could be considered examples of involuntary actions that are similar to reflexes. The neuroanatomy has been linked to lesions in the frontal lobe, and to our knowledge nothing has been done so far in terms of brain coordinated activity. Nevertheless, a possibly related study by van der Helden et al. assessing brain coherence during observational learning (that is, subjects had to imitate movements) found an enhanced fronto-parietal coherence in these imitation tasks (van der Helden et al., 2010). Whether this observation indicates that individuals affected with environmental dependency syndromes display more synchrony in their brains during the impulsive behaviors cannot be assured at this point due to lack of studies, but that hypothesis opens up an intriguing possibility.

## Involuntary movements in parkinson disease

The motor dysfunctions present in Parkinson disease (PD), and other deviations of motor coordination in Huntington's disease and tardive dyskinesia, provide another illustration of how changes in organized patterns of activity of the nervous system lead to deviations of normal and adaptable function, specifically to involuntary movements characterized in these cases by tremors and other actions.

For the sake of brevity, let us state in simple terms that the (main) molecular basis of this disorder is the degeneration of the dopaminergic neurons of the substantia nigra, which causes a dysfunction in the areas to where these cells project. The resulting altered activity in these areas show a tendency to synchronization: as dopaminergic transmission decreases, neurons in the subtantia nigra become more excitable to cortical inputs and there is an enhancement of synchronous activities in the nigral and basal ganglia-cortical networks. This conclusion stems from evidence obtained in rodent models of PD where the dopaminergic fibers have been damaged, using 6-OHDA which is toxic to dopaminergic cells. This revealed a tendency toward hyperexcitability in the substantia nigra pars reticulata and the consequent enhancement of rhythmic synchronization between the basal ganglia and cortical networks after dopamine depletion, and, more specifically, the response of basal ganglia cells to cortical inputs was found to be enhanced after the dopaminergic lesion (Belluscio et al., 2007; Dejean et al., 2008). Evidence for enhanced tendency to synchronized activities in a diversity of cortical areas during pathological tremors has been obtained too at the level of MEG or scalp EEG (Volkmann et al., 1996; Tass et al., 1998). Indeed, the success of deep brain stimulation (DBS) in halting Parkinsonian tremor is derived from its net effect, which is an effective reduction of activity in key structures thus reducing excitability and thereby opposing synchronizing tendencies.

Interestingly, similar reduction in tremor occurs after lesions of the subthalamic nucleus (subthalamotomy) which may seem paradoxical on a cursory look, as the irreversible damage to the subthalamic nucleus causes same effect as its high-frequency stimulation in DBS, but the net effect in both cases is the same: decreased excitability in connected regions particularly the globus pallidus. Hence in the final analysis what is found is an altered coordination dynamics in basal ganglia-thalamo-cortical circuitries, resulting from hyperexcitability and enhanced synchrony in restricted brain populations whose manifestations are will-less actions specific to the nature of those nervous circuitries involved.

## Tics in tourette syndrome

Tourette syndrome provides further examples of involuntary actions, known as tics. The syndrome is characterized by motor and vocal tics, as well as psychiatric co-morbidities. Is there evidence for a disrupted coordinated activity and particularly higher synchronization between brain areas associated with tics? A direct computation of brain synchrony patterns during tics in this syndrome has been done in one study so far (to the author's knowledge), but some studies, mostly based on neuroimaging data, have investigated the brain circuits whose activity correlate with the generation of tics in Tourette's syndrome. Thus, a variety of regions have been detected to become more, or less, active during tics and in other various situations such as movement execution (e.g., finger tapping) with the general finding that, perhaps unsurprisingly, brain activity is organized differently in the patients as compared with control participants (Biswal et al., 1998; Bohlhalter et al., 2006; Church et al., 2009). In the study where synchrony was more directly assessed, Serrien et al. estimated coherence at the alpha band (8–12 Hz) derived from EEG scalp recordings and found enhanced coherence between prefrontal and mesial cortex and sensorimotor regions, but this was estimated during the voluntary suppression of tics rather than during the involuntary tics. The conclusion of the authors was that frontomesial networks become overactive in patients (Serrien et al., 2005). However, whether same elevation of coherence occurs during tics remains to be addressed. Franzkowiak et al. (2010) used event related synchronization and desynchronization but these measures are not directly related to synchronization as denoted in our work.

There are further indications, albeit somewhat indirect due to the data used and the analyses performed, of increased synchrony between specific brain regions. Using Granger causality to assess what is normally labeled as directionality of coupling, and derived from fMRI data, Tourette's syndrome individuals exhibited stronger neural activity and interregional causality than healthy subjects throughout portions of the motor pathway, including the sensorimotor cortex, putamen, pallidum, and substantia nigra, and the activations were stronger during spontaneous tics as opposed to “voluntary” tics (Wang et al., 2011). Futhermore, this study reported decreased brain activity in the patient group in some cortico-thalamic pathways that may exert top-down control over motor actions.

As another suggestion of higher-than-normal synchronous brain activity associated with this condition, enhanced “connectivity” using independent component analysis of fMRI data was found in some patients as compared to controls during performance of tasks such as finger tapping (Werner et al., 2011). In another fMRI study, the derived connectivity patterns differed between patients and age-matched controls. More specifically, patients displaying stronger local connections while weaker distant fronto-parietal connections, thus indicating that some “control networks” (at least in terms of executive functions) in paediatric Tourette syndrome have immature and anomalous patterns of functional connectivity (Church et al., 2009).

That there could be abnormally elevated excitability in some neuronal networks in this syndrome has been believed for some time. Specifically, the local cell populations in the striatum become more active in patients thus resulting in disinhibition of the thalamocortical circuits and the generation of tics. This chain of neurophysiological events is due to the particular cell types and connections in the complicated basal ganglia circuitries. In this regard, only one study has recorded directly cell activity during tics, study that had to be performed in monkeys as Tourette patients are not invasively studied (with implantation of intracranial electrodes). This study found that tics in the monkeys (induced by local disinhibition of the putamen using the GABA_A_ receptor antagonist bicuculline) were associated with brief bursts of action potential firing in the putamen, and subsequently, activation of cortical areas were detected; however, the activity of the basal ganglia areas (globus pallidus) traditionally thought to be the precursors of the abnormal cortical activity appeared after tic-related cortical activations (Bronfeld et al., 2011). Therefore, the globus pallidus may not initiate tics (at least in these monkeys, of course the situation can be different in patients or in other tics unrelated to the syndrome), but still these studies furnish indications of local higher spiking activity through striatal-cortical chains and increased synchrony associated with tics.

Tics and the previously addressed Parkinsonian movements are to a large extent irrepressible actions, the main difference at the cognitive level is that the former are accompanied by an urge to perform the movement whereas in the later the actions occur without urges. At the neurophysiological level, it can be conjectured that the reason for that difference is due to the widespread activation in case of tics that involve higher-order association areas and thus the “cognitive” component appears as an urge in the individual's mind. Recall the aforementioned conclusion by Serrien et al. (2005) that frontomesial networks become overactive in Tourette's patients, and the study in monkeys showing that basal ganglia areas become active after tic-related cortical activity (Bronfeld et al., 2011). On the other hand, the Parkinsonian movements seem to materialize mostly from the synchronous activities in basal ganglia, thalamic, and motor cortical regions. The fewer higher-order association areas that are involved in an action, the more likely the action will be/feel involuntary, which is supported by the observations made in patients where direct motor cortex stimulation causes actions but these remain out of awareness (see comments in section “The Function of Brain Coordinated Activity in Sensing Volition”).

## Altered brain coordinated dynamics during hypnosis and different types of dreams

To continue this brief route over the correlations between brain dynamic patterns and apparent unintentional behaviors, hypnotic suggestions offer possible examples of involuntary actions, perhaps similar in nature to those of the alien hand syndrome aforementioned in the sense that the actions may feel intentional to the hypnotized subject but yet, be outside of his/her will. It is not easy to put together the results of the few studies that have assessed coherence/connectivity during hypnotic states because each used different experimental (behavioral) methods (and of course analytical, but this is to be expected considering the multitude of techniques to quantify synchronization) and focused mostly on comparing high versus low susceptible/suggestible individuals. If some commonality amongst the observations of those studies can be emphasized, it is that of altered synchrony brain patterns during hypnosis, with some studies reporting increases and other decreases in synchrony depending on subjects and location of sensors (Egner et al., 2005; Fingelkurts et al., 2007; White et al., 2009). Most of these few reports focused on measuring frontal and parieto-frontal coherence, and in some cases significant alterations were found during hypnotic states which indicates that, similar to the case of dreaming addressed below, altered fronto-parietal coordination is associated with the performance of hypnotically suggested acts.

To close this section, perhaps a brief comment on the brain activity patterns during dreams may be of relevance, specifically comparing normal dreams, those with “actions” without much will control, and lucid dreams, in which the actions are performed with a higher degree of self-awareness and insight: the dreamer is aware that he/she is dreaming and therefore can deliberately influence the actions. Hence the question can be asked as to what differences in brain dynamics there could be between lucid and normal dreams. Could there be fewer fluctuations in coordinated activity (that is, more constant widespread long-lasting synchrony) in normal dreams as opposed to lucid ones? Not much is known, but evidence indicates that in normal, will-less dreams, the coordination between parietal and frontal cortices is lost or at least less than in lucid dreaming. Specific analysis has revealed more beta band activity in parietal cortical regions during lucid compared with non-lucid dream (Holzinger et al., 2006) and coherence amongst frontal sensors, derived from scalp EEG recordings, was found to be higher in lucid dreams than in non-lucid ones and similar to the coherence in waking states (Voss et al., 2009).

Thus, while not much has been investigated yet, the picture that emerges is that lucid dreaming maintains similar brain coordination dynamics as waking states especially with involvement of widespread distribution of activity/information along parietal and frontal areas. This is not too surprising as these cortical regions are thought to be fundamental in the performance of executive functions and self-awareness. No studies so far have addressed and compared the variability and fluctuations of the brain coordination patterns during both types of dreams hence nothing can be said, but the aforementioned evidence indicates that the loss of particular coordination dynamics in frontal and parietal regions is associated with non-lucid, will-less dreams.

## The function of brain coordinated activity in sensing volition

The direct investigation of the optimal patterns of organized activity that produce deliberate actions is not a trivial matter because experiments are difficult to perform. Ideally, direct stimulation of selected brain areas and the subsequent determination of the spread and coordination of the imposed activity are needed to directly address these matters. In this regard, some studies performed on a variety of patients with intracerebral electrodes that were undergoing awake brain surgery are of relevance. The results obtained with these individuals present evidence for the importance of widespread activation of several brain regions in voluntary actions. The main results of these studies revealed specific cortical areas that process information related to the intention to perform movements and the awareness of the motor actions (reviewed in Desmurget and Sirigu, 2009), and shed light on what possible coordination of activity between cortical areas are necessary for the experience of intentional movements (for a review on brain networks involved in voluntary actions, see Haggard, 2008).

Parietal lobe stimulation in these patients induced a will to move but without actual action performance, and increasing the stimulation intensity resulted in patients claiming they had produced a movement that was never done. This was probably the effect of activating connected areas in addition to the parietal due to higher intensity of stimulation. Thus, more widespread activation may lead to erroneous perception (sense of volition/motions) if the coordination (synchronization) between those local areas that were stimulated and the connected regions is not optimal. But, as there has not been any synchrony assessment in these studies, this remains speculation.

On the other hand, frontal cortex stimulations (premotor cortical areas) evoked actions but these were unconscious, as patients denied (unless offered visual feedback) performing the movements. Hence motor awareness seems to emerge from predictions made about the movements rather than from the sensory feedback (propioception) caused by the movement itself. These results underline the relation between the interaction (that will be reflected in coordination of activity) in fronto-parietal areas and the expression or feeling of volition. These two fundamental cortical regions are implicated in executive functions and there is an extensive literature on this subject. The possibility to change the stimulation intensity in this experimental setting makes it possible to investigate how more brain areas may become recruited in movement awareness/monitoring as the stimuli delivered through the intracerebral electrodes become larger.

Alterations of brain waveforms are indicators as well of different coordination dynamics associated with these phenomena. For instance, the observation that patients with posterior parietal damage do not display normal readiness potentials (made famous by Benjamin Libet but originally described by Kornhuber and Deecke, 1965) prior to voluntary movements (or even during observation of actions, Fontana et al., 2012) is another indication of a lack of normal coordination that results in altered recorded waveforms. Furthermore, there is neuroimaging evidence in vegetative and minimally conscious state patients that underscore the association of widespread brain activation with the level of consciousness (Schiff et al., 2005; Schiff, 2010). Combined together, all these data obtained in these patients indicate several brain areas that can be part of a network responsible for, or associated with, the sense of agency. Interactions amongst parts of this network could be scrutinized, as recently proposed by David (2012), using dynamic causal modeling.

Please note that in spite of the emphasis that has been placed here on the notion of the importance of widespread coordinated activity associated with awareness and volition, I am not implicating the need to be conscious/aware of decision-making for actions to be “free” (Heisenberg, 2009), but rather to experience “free will.” The perception, or experience, of the freedom of the will by individuals can be scientifically studied, unlike the intrinsic concept of freedom which is more a subject for philosophy.

## Concluding remarks

The evidence here reviewed suggests that altered synchrony perturbs the transient coordination dynamics of brain cell ensembles, such that automatisms or reflexes that are imprinted in the circuitries may emerge and remain out of consciousness thereby becoming involuntary. Still, what is in the transient synchrony patterns that allow “will” to be sensed? These patterns are reflections of the coordinated widespread activity in various brain areas needed for the integration and segregation of information associated with cognition. Sensory inputs are segregated and processed by distinct brain modules (visual, auditory…) and later have to be integrated in association areas like frontal or parietal cortices. Perhaps in one of those areas, (the prefrontal being a candidate), there exists a “sixth” sense that perceives not the external sensorium but the internal brain activity, and in this sense consciousness and related aspects like free will become perceptions, as denoted above when discussing the alien hand syndrome. As E. T. Rolls puts it: “… if a system were doing this type of processing (thinking about its own thoughts), it would then be very plausible that it should feel like something to be doing this” (Rolls, 1999).

**Figure 1:**
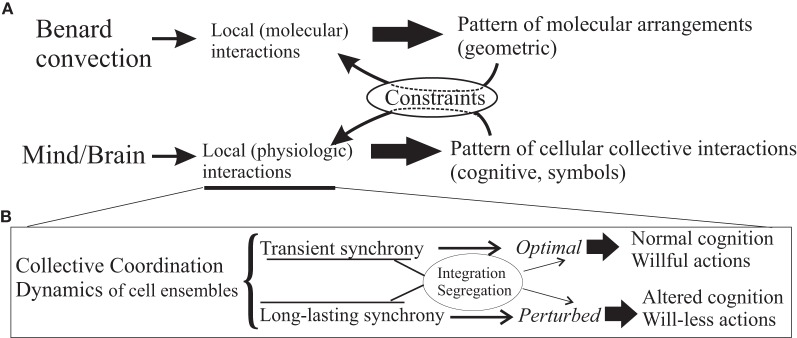
**Emergent mind/body phenomena.** Panel **(A)**, simple scheme illustrating that mind/body phenomena can be considered as emergent properties of the whole system derived from the local physiologic characteristics, fundamentally interactions amongst cells, as much as the local molecular interactions in silicone oil give rise to the geometric patterns of the well-known Bénard convection. These emergent patterns, in turn, act on and modulate/constrain the interactions amongst the system's constituents (or, in the language of physics, the order parameter feeds back on the control parameters). In the case of brains, the patterns can be thought of as the symbols with which at a certain, cognitive level the brain operates, these symbols being representations of some aspects of the reality experienced by the organism that has a brain. As remarked in the text, intentions being high-level cognitive events or “symbols,” can be conceived to act as constraints of the dynamics. In panel **(B)**, a schematic summary of the main message in the text, or how the local physiologic interactions can give rise to aberrant coordinated activity depending in part on the synchronous activities in specific brain cell ensembles that result in altered cognitive processes and behaviours.

It can be said that, at a high level of description, brains operate with (cognitive) symbols, reflection, and manifestation of the neurophysiological activity of the whole organ, which in turn act on those neurophysical events (Figure 1). For these reasons, clear-cut answers to the question of whether conscious intention “causes” behavior are hard if not impossible to find. It is customary to declare that intention “causes” brain activity that produces actions, and a common interpretation of the famous Benjamin Libet's experiments is that the brain has already made a choice some milliseconds before “we” become aware of that decision, as if there were different people inside a brain. If these dichotomies are surmounted, the problem in the interpretation of the Libet experiment is significantly reduced, interpretation which was in fact already given by Libet himself: “unconscious cerebral processes precede a subjective sensory experience” (Libet, 2006); this latter sensory experience is the perception of the will. What events enter consciousness depends on what brain areas share those specific contents and this spread of activity will determine whether or not they become conscious (the global workspace theory aforementioned). Indeed many aspects of the willed and well-articulated movements do not enter consciousness, as we are not aware of the precise motion of specific muscles to achieve certain goal; only the general course of action enters awareness and is described as voluntary. The reason for this apparent dissociation of lack of awareness of specific motor actions and the general, global sense of will, could be that some activity is not broadcast to widespread brain regions but remains restricted to local neighborhoods (for example in primary motor cortex), while other activity patterns (encoding/representing goals) reach certain association areas and enter consciousness/awareness. Evidence for the importance of widespread activation of several brain regions in voluntary actions was emphasized above.

These here proposed biophysical signatures of wilful actions can be viewed from a higher-level, more abstract but equivalent perspective using dynamic system frameworks, as for instance in the work of Albantakis and Deco (2011), in that decision or choice making is described in a state space with attractor networks. After all, voluntary actions are not taken from an infinite set, rather the whole state space, in this view, is not fully searched but only a few states, attractors that represent choices, are selected, which is a feature imposed, probably and at the physiological level, by the modularity of the nervous system (modularity in biological systems reduces the search of the dynamic state space so those that are more adaptive and become the preferred solutions, see Lorenz et al., 2011). Viewing cognitive phenomena as dynamic structures, and invoking the principles of self-organization and emergence, consciousness along with its features can be seen as emergent properties of the nervous system, as much as the geometric pattern in the Bénard convection (Bergé et al., 1984) results from the properties of the oil and its environment, schematized in Figure 1 (similar arguments have been recently made by Coey et al., 2012). Within this framework, intentions have been conceptualized as constraints in the emergent coordination, rather than causal entities of behaviors, thus transcending aspects of the mind/body “problem” (Juarrero, 1999; Kloos and van Orden, 2010) which, incidentally, it is my opinion that this is not a problem but rather, an ill-posed question. Some authors have proposed a complementary science of brain, mind, body and behavior, and remarked the universal and seemingly unavoidable tendency to dichotomize and the troubles derived from it (Kelso, 2008). In a century characterized by the rise of complexity science, transcending dichotomies should be easier than ever. For some scientists, like Wolfgang Pauli (1955), “It would be most satisfactory of all if physics and psyche could be seen as complementary aspects of the same reality” (in a lecture given in 1948 at the Psychological Club of Zurich).

### Postscript: on randomness and determinism in behavior

The evidence reviewed, especially with regards to the emphasis placed on synchronization of cellular activity as determinants of voluntary and will-less actions depending on the degree and “quality” of the synchrony, may be considered as suggesting a determinism in behavioral actions, as the spike firing synchronization of a population of neurons imposes a certain forcing on the activity of the connected cells (synchronous arrival of synaptic inputs on a neuron increases its firing probability), the next stage in the chain that transmits the neural signals as action potentials. Is there room for randomness, or indeterminacy, in behavior or is this “forcing” from cell network to cell network making all actions purely deterministic? This is a matter that can be discussed at great length and thus not appropriate here, perhaps only to declare my agreement with the words pronounced by the mathematician Kurt Gödel that, to me at least, seem to nearly put an end to further discussion on this topic of determinism versus “free” will: “there is no contradiction between free will and knowing in advance precisely what one will do. If one knows oneself completely then this is the situation. One does not deliberately do the opposite of what one wants” (in Rucker, 1995). As well, paraphrasing Einstein when he talked about space and time, it can be said that order and randomness are modes by which we think and not conditions in which we live. Behavior and brain function can be described as *stochastic*, because of the unfeasibility to know all factors, mental or environmental, involved in producing actions. However, *randomness* implies a more intrinsic indeterminacy independent on the observer, and much can be debated about this topic.

### Conflict of interest statement

The author declares that the research was conducted in the absence of any commercial or financial relationships that could be construed as a potential conflict of interest.
